# Cryogels and Monoliths: Promising Tools for Chromatographic Purification of Nucleic Acids

**DOI:** 10.3390/gels10030198

**Published:** 2024-03-14

**Authors:** João Ribeiro, Marco Â. Luís, Bruno Rodrigues, Fátima Milhano Santos, Joana Mesquita, Renato Boto, Cândida Teixeira Tomaz

**Affiliations:** 1 CICS-UBI—Health Sciences Research Centre, University of Beira Interior, 6201-506 Covilhã, Portugalb.rodrigues99@gmail.com (B.R.); joanamesquita3@gmail.com (J.M.); rboto@ubi.pt (R.B.); 2 Department of Chemistry, University of Beira Interior, Rua Marquês de Ávila e Bolama, 6201-001 Covilhã, Portugal; 3 Functional Proteomics Laboratory, Centro Nacional de Biotecnología, Consejo Superior de Investigaciones Científicas (CSIC), Calle Darwin 3, Campus de Cantoblanco, 28049 Madrid, Spain

**Keywords:** chromatography, cryogels, monoliths, pDNA, purification, RNA

## Abstract

The increasing demand for highly pure biopharmaceuticals has put significant pressure on the biotechnological industry to innovate in production and purification processes. Nucleic acid purification, crucial for gene therapy and vaccine production, presents challenges due to the unique physical and chemical properties of these molecules. Meeting regulatory standards necessitates large quantities of biotherapeutic agents of high purity. While conventional chromatography offers versatility and efficiency, it suffers from drawbacks like low flow rates and binding capacity, as well as high mass transfer resistance. Recent advancements in continuous beds, including monoliths and cryogel-based systems, have emerged as promising solutions to overcome these limitations. This review explores and evaluates the latest progress in chromatography utilizing monolithic and cryogenic supports for nucleic acid purification.

## 1. Introduction

In recent times, the demand for new biopharmaceuticals has increased at an unprecedented rate, prompting the biotech industry to create cutting-edge technologies for the production and purification of these bioproducts [1]. These advancements hold paramount importance for various applications, including gene therapy, vaccines [2], protein replacement therapy, immunotherapy, and cell reprogramming, among others [3]. Additionally, these applications require a significant quantity of biopharmaceuticals characterized by high purity and quality to meet the stringent standards set by regulatory agencies, such as the Food and Drug Administration (FDA) [4]. Producers must demonstrate the absence of adverse effects on the identity, strength, quality, purity, or potency of biotherapeutics to ensure product safety and effectiveness. Impurities in biotherapeutic products, such as host cell proteins, can trigger immunogenic responses that may impact the safety and effectiveness of the product, highlighting the importance of removing impurities to prevent adverse reactions and to safeguard the product from degradation and maintain its therapeutic properties [2].

The purification of nucleic acids, such as plasmid DNA (pDNA), minicircle DNA (mcDNA), messenger RNA (mRNA), small interfering RNA, and microRNA, presents inherent challenges due to the unique physical and chemical characteristics of these compounds. In fact, nucleic acids are highly sensitive molecules susceptible to degradation by nucleases, heat, or pH changes. Therefore, meticulous handling is imperative throughout the purification process to maintain the integrity, stability, and functionality of nucleic acids. Additionally, achieving high purity and yield during nucleic acid purification entails overcoming challenges associated with separating them from contaminants such as proteins, lipopolysaccharides, and small RNAs, which can adversely affect the quality of the final product [4]. Consequently, the intricate physical and chemical properties of nucleic acids necessitate sophisticated purification methods to effectively isolate them from complex biological samples, thereby adding complexity to the purification process [1].

Currently, nucleic acids can be purified from cell extracts using various specialized procedures, with chromatographic techniques being the most common [4]. Liquid chromatography, in particular, finds extensive application at analytical, preparative, and industrial levels owing to its versatility, cost-effectiveness, robustness, and high reproducibility. These benefits make chromatography a widely used method in laboratories for efficient and reliable nucleic acid purification processes. In fact, different chromatographic techniques, including affinity chromatography (AC) [5], hydrophobic interaction chromatography (HIC) [6], size exclusion [7], and ion exchange chromatography (IEX) [8] have been employed. Despite the advantages of conventional chromatography for nucleic acid purification such as rapidity, high purity, ease of use, automation capabilities, and versatility these techniques commonly use particulate matrices such as agarose, dextrose, or silica, which present drawbacks such as low flow rates and losses at high pressures [9]. Moreover, they exhibit low binding capacity and high mass transfer resistance for the movement of large biomacromolecules like pDNA and mRNA [10].

To overcome these limitations, several supermacroporous supports have been developed, allowing the formation of convective channels within the supports to minimize mass transfer resistance [11]. Despite these efforts, a degree of diffusion transport is still observed in the pores of these systems [12].

**Figure 1:**
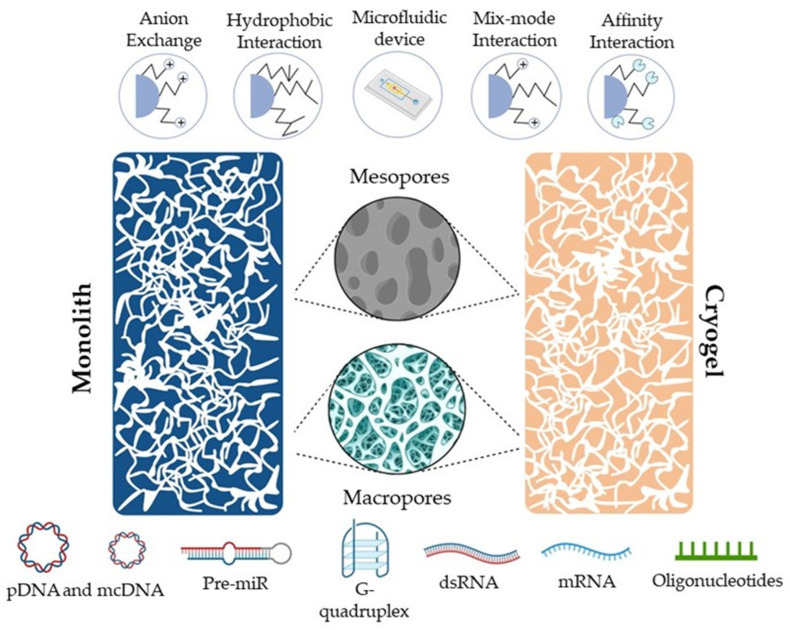
Mesopores and macropores in monoliths and cryogels are pivotal for nucleic acid purification via chromatography. Monoliths: mesopores amplify surface area for efficient molecule interaction, boosting purification efficacy and macropores serve as fluid conduits, easing solvent transport and minimizing mass transfer resistance. Cryogels: the interconnected macropores offer minimal pressure drop, short diffusion path, and retention time, facilitating rapid biomacromolecule enrichment and purification.

In recent years, the development of continuous beds, such as monoliths and cryogel-based systems, have provided alternative solutions to address the flux limitations of diffusion transport inherent in conventional chromatographic matrices. Monoliths and cryogels offer higher external porosity compared to particle-based supports, resulting in increased permeability and lower back pressures in chromatographic systems, thereby enhancing the efficiency of nucleic acid purification processes. Additionally, their unique structure of interconnected macropores enables high flow rates, due to convective transport, allowing the processing of large sample volumes in a short time frame, thus improving the speed and throughput of the purification process (Figure 1).

Furthermore, monoliths and cryogel-based systems also overcome the limitations of conventional chromatography by offering versatility in formats and selective separation capabilities, thereby enhancing nucleic acid purification efficiency and specificity.

This review provides a comprehensive description and discussion of the progress achieved in chromatographic processes employing monolithic and cryogel supports for the purification of nucleic acids.

## 2. Monoliths

Monoliths represent the fourth generation of chromatographic matrices, characterized by a unique structure featuring highly interconnected channel networks making them distinct from other supports [13]. Although first introduced in the 1990s [9], monoliths have gained prominence as alternatives to traditional packed columns [14]. In fact, the diverse channels of the monolith [15] offer a high porosity with both mesopores and macropores interconnected in a multitude of configurations [16,17]. Mesopores afford ample surface area for molecules to interact with the matrix, whereas macropores serve as conduits for solvent transportation [18] (Figure 1). These characteristics endow monoliths with low mass transfer resistance and ease of functionalization and preparation. Consequently, monoliths improve the interaction with target molecules, enhancing the purification efficiency when compared to conventional supports [18]. Monoliths can be prepared using different types of substrates, so they can be classified into various subcategories such as organic, inorganic, or hybrid [19,20].

Organic monoliths, as the name suggests, are crafted using a combination of organic monomers, an initiator, crosslinkers, and a porogenic solvent [21]. Commonly used monomers in the synthesis of organic monoliths include methacrylates [22], acrylamides [23], and styrene [24]. Solvents like propane-1-ol and butane-1,4-diol facilitate homogeneous mixing to control column porosity [25]. Crosslinkers act as bridges between polymeric chains, enabling multi-directional growth. These compounds, featuring one or more double covalent bonds, allow carbon chain branching during polymerization [25]. The reactions can be induced by heat, by light emitting diode light, ultraviolet radiation [26], or by chemical induction through the use of N,N,N,N-tetramethylethylenediamine (TEMED) and ammonium persulfate (APS) [27]. Organic polymer monoliths are typically compatible with extreme pH conditions, have good biocompatibility, are easily synthesized, and exhibit high interconnectivity. However, they are prone to swelling, shrinking, and instability temperatures higher than 200 °C [28]. Organic monoliths feature large pores suitable for macromolecule separation.

Inorganic monoliths predominantly consist of various inorganic materials, including alumina, hydroxyapatite, and silica, among others, with silica being the most prevalent [29]. Typically, these supports are synthesized through a sol–gel technique employing different procedures tailored to the specific inorganic material [29,30,31,32,33]. The compounds undergo hydrolytic polymerization in an aqueous solution containing acetic acid and polyethylene glycol (PEG), and subsequently, ammonia treatment is applied to generate mesopores within the silica structure [34]. Inorganic monoliths demonstrate enhanced resistance to organic solvents and mechanical stability. Nonetheless, similar to all silica-based supports, they are limited to a pH range of 2 to 8, and their preparation process may pose challenges in terms of control [35].

Hybrid monoliths, alternatively referred to as organic–inorganic hybrid monoliths, consist of two or more constituents combined at a nanometric or molecular level [35]. They are classified according to their chemical composition into hybrid silica-based monoliths and hybrid polymer-based monoliths. Hybrid polymer-based monoliths are prepared via matrix functionalization through chemical bonding, allowing the preservation of specific structures for the functional groups [35,36]. On the contrary, silica-based monoliths are synthesized via the sol–gel process utilizing a silica precursor with organic moieties [37]. These supports offer remarkable biocompatibility, good mechanical properties, flexibility, and extended lifetimes [38]. Furthermore, leveraging the advantages of both organic and inorganic monoliths, hybrid supports have attracted interest due to their improved characteristics, such as high surface area, elevated sensitivity, and excellent thermal stability [36].

### 2.1. Monoliths for Nucleic Acid Purification

**Figure 2:**
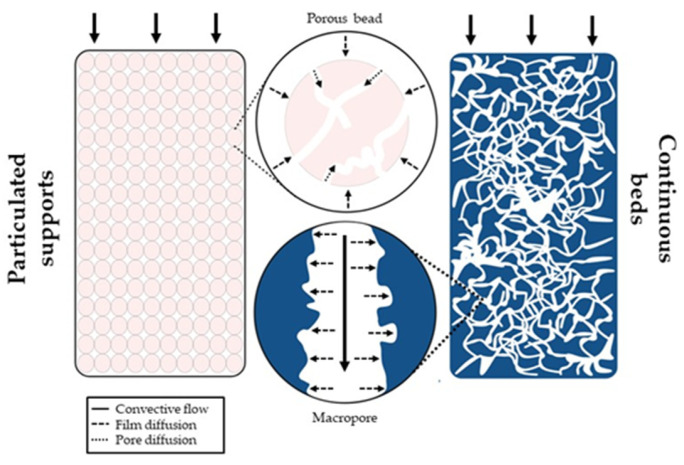
Schematization of transport phenomena by diffusion in a packed-bed chromatography column (particulate support) and by convective flow in continuous beds (cryogels and monoliths). The diffusive mass transport is related to a slow process, lower resolution, and low capacity. In convective mass transport, the big channels allow laminar flow with no shear forces, leading to a flow-independent resolution and capacity.

The purification of nucleic acids is jeopardized by the low performance of commercially available chromatographic matrices, predominantly those based on highly porous particles. The particulate matrices were designed to have a high adsorption capacity for proteins, featuring a small pore diameter, tailored for the size of proteins, which is typically smaller than that of nucleic acids [39]. Consequently, in conventional matrices, molecules with larger diameter, such as pDNA or mRNA, only adsorb on the outer surface of the beads [10]. This results in significantly lower adsorption capacity compared to that observed for proteins. On the other hand, traditional liquid chromatography operates as a slow and diffusion-controlled process, posing challenges in purifying unstable biomolecules that require a fast and efficient separation process. Monoliths have emerged as a viable alternative to address the constraints associated with the diffusion of these molecules or their binding capacity (Figure 2). Therefore, this type of support increasingly stands out as an important standard in the purification of nucleic acids, especially pDNA.

#### 2.1.1. Monoliths for DNA Purification

In recent years, several monolithic supports based on chromatography modes, including IEX, HIC, and AC, have been applied to DNA purification. A summary of the monolithic supports used in the several studies detailed below is presented in Table 1.

##### Ion Exchange Chromatography Using Monoliths for DNA Purification

IEX is a versatile and powerful technique for nucleic acid purification, offering high resolution and the ability to process large sample volumes. Anion exchange chromatography (AEX) is commonly employed for pDNA purification, taking advantage of the fact that the negatively charged phosphate groups of nucleic acids interact with positively charged ligands in the stationary phase. Although lysates contain different topologies with similar overall charge and molecular weight, differences in the conformation of the DNA isoforms allow their separation based on the distribution of the charge densities, [65]. The choice between bead-based supports and monoliths depends on specific application needs. Bead-based supports provide flexibility with a broad range of ligands and established protocols, whereas, monoliths offer advantages like high flow rates and reduced pressure drop, making them ideal for applications prioritizing speed and throughput. Decision factors include resolution, selectivity, cost, and the scale required for the nucleic acid purification process.

One of the pioneering publications on the application of monolithic columns for DNA purification was presented by Forcic et al. (2005). They targeted genomic DNA (gDNA) from bacterial and mammalian cells lysed through alkaline lysis and TritonX-100-based lysis, respectively. A monolith employing diethylaminoethyl (DEAE) as a ligand, which imparted weak anion exchange characteristics to the support, made it possible to effectively separate and purify gDNA directly from the lysate without additional manipulations. The purity was further confirmed by electrophoresis, where a sharp gDNA band was observed in the elution fraction free from RNA contamination [40].

Urthaler et al. (2005) performed one of the first works utilizing AEX and convective interaction media (CIM^®^) monoliths for pDNA purification at a larger scale to achieve a pharmaceutical-grade plasmid. Two pDNAs of different sizes, 4.9 kbp and 6.9 kbp, obtained from a clarified alkaline *E. coli* lysate and pre-purified lysates with a HIC capture step, underwent a screening with weak anion exchangers DEAE, ethylenediamine (EDA), and the strong anion exchanger quaternary ammonium (QA). DEAE demonstrated a resolution of 1.31, nearly triple that achieved with QA. It was also significantly higher than EDA, which failed to resolve the peaks. DEAE also achieved the highest recovery (100%) and estimated purity (92%) of the covalently closed circular DNA isoform. CIM^®^ DEAE exhibited the highest binding capacity, independently of the flow, reaching an impressive 8.86 mg/mL, surpassing conventional bead supports (3.29 mg/mL) at the same flow rate. The laboratory-scale experiments were scaled up using three pre-packed CIM^®^ DEAE monoliths (8 mL, 80 mL, and 800 mL), maintaining reproducibility and robustness in all cases. Despite traces of host protein still being detected in the eluted samples, the purified pDNA complied with safety and quality regulations [39].

Ongkudon et al. (2011) introduced a novel ion exchange conical monolithic support using diethylamine- and triethylamine-activated polymethacrylate for processing a plasmid-based measles vaccine from a clarified alkaline *E. coli* lysate. These two amino-based ligands, chosen for their low positive surface charge densities and capacity to mitigate wall channeling, underwent functionalization to achieve a conical chromatographic support with exceptional durability and minimal side flow. Dynamic binding capacity studies yielded 21.54 and 15.78 mg pDNA/mL support for the diethylamine- and triethylamine-activated monoliths, respectively. Following optimization of experimental conditions, triethylamine support achieved the best results in the depletion of host impurities, while maintaining pDNA recovery above 95%. Despite high pDNA binding and near-ideal recovery, further studies were recommended to meet Food and Drug Administration (FDA) safety regulations [66], specifically concerning the presence of host protein in the purified samples [41].

As an analytical approach, Cernigoj et al. (2021) investigated IEX monoliths for the separation of pDNA isoforms using guanidine chloride as the elution buffer. Guanidine weakens hydrophobic and electrostatic interactions, disrupting hydrogen bonds between pDNA and the monolith. DEAE and QA monoliths were tested, revealing that guanidine did not enhance the elution performance of the QA monolith. For the DEAE monolith, the elution using exclusively guanidinium chloride significantly improved resolution (7.8 vs. 4.4 with sodium chloride [NaCl]) but decreased recovery levels, which were addressed by combining guanidinium and NaCl. This adjustment maintained the high resolution but achieved nearly 100% recovery [42].

More recently, guanine quadruplexes (G4s) have drawn significant attention from the scientific community, standing as crucial structural motifs in nucleic acids with pivotal roles in DNA replication, gene expression, genome stability, and transcriptional regulation. Addressing the demand for highly purified G4s, Kazarian et al. (2020) introduced a method employing a CIM^®^ monolith with a strong anion exchanger (QA) [43]. Following process optimization, a well-resolved peak was obtained corresponding to the G4 formed from single-strand oligonucleotides within the mixture. This finding was corroborated by mass spectrometry, which exhibited a prominent G4s peak, corresponding to 92% purity. The authors demonstrated an effective purification method capable of obtaining a pure G4s sample from a complex mixture of single-strand oligonucleotides and partially formed and fully formed G4s, while maintaining a binding capacity of 5.5 mg/mL [43].

##### Hydrophobic Interaction Chromatography Using Monoliths for DNA Purification

HIC has garnered significant attention for purifying pDNA and its isoforms due to variations in the hydrophobicity of the biomolecules within bacterial host cells. The purification process by HIC relies on the use of high salt concentrations in mobile phase buffer during initial equilibration conditions, thus strengthening the interaction between pDNA and the hydrophobic support while removing less hydrophobic contaminants in the flow-through [67].

In 2011, Sousa et al. [44] demonstrated the efficiency of a carbonyldiimidazole (CDI) monolith in the purification of supercoiled (sc) pDNA isoform from *E. coli* lysates, employing the same salt and ionic strength conditions that were previously applied in a histidine-based chromatographic strategy [68,69]. In both cases, the ligands contained an imidazole group, which is known for its high specificity and affinity for the biological active sc isoform, which exhibits higher biological activity than the open circular (oc) isoform. The HPLC analysis of the fraction purified using the CDI monolith revealed a 100% purity, a yield of 89%, and a step recovery yield of 74% for sc pDNA isoform. Moreover, the maximum impurity levels detected complied with FDA specifications [66]. The DBC of CDI monolith for pDNA was 6 to 12 times higher when compared with histidine agarose-based supports [69], indicating their significant potential in sc pDNA purification. The chromatographic behavior of these nongrafted CDI monolithic disks was also successfully tested for the separation of purified pDNA of different sizes (14 kbp, 10.292 kbp, and 2.686 kbp) under hydrophobic conditions [70]. Furthermore, the impact of ammonium sulfate (NH_4_)_2_SO_4_ concentration on the eluent and the applied flow rate were evaluated. The optimal binding capacity was achieved at lower flow rates and with higher salt concentrations, independently of pDNA size. A decrease of over 95% in the binding capacity was observed by slightly reducing the concentration from 3 M to 2.5 M (NH_4_)_2_SO_4_, highlighting the importance of the high salt concentration for achieving the ideal DBC. The effect of the pH on DBC was also assessed, indicating that pDNA bounds more efficiently at pH = 8, resulting in a maximum DBC of 5.891 mg pDNA/mL support with 3M (NH_4_)_2_SO_4_. The effect of pH on DBC can be explained by the fact that the pKa of the histidine imidazole ring is 6.5. As the pH increases above this value, it acquires a more positive global charge, enhancing interaction with the negative charge of the pDNA, thus promoting higher binding capacity [70].

Sample displacement chromatography (SDC) was employed by Cernigoj et al. (2015) to separate pDNA isoforms under overloading conditions, where sc isoform acted as a displacer of other isoforms. In this study, three CIM^®^ monoliths with distinct hydrophobicities (CIM^®^ C4 HLD, CIM^®^-pyridine, and CIM^®^-histamine) were used for pDNA isoforms separation. CIM^®^ pyridine monolith was produced by modifying a CIM^®^ epoxy monolith with a solution of 2-mercaptopyridine, while the CIM^®^ histamine was synthesized by immobilizing histamine in a CIM^®^ CDI monolith. After experimentation, researchers observed that the CIM^®^ C4 HLD monolith presented the highest DBC_10%_ for sc isoform, confirming the selectivity for this isoform since the DBC_10% sc pDNA_ was 12 times higher than the DBC_10% oc pDNA_. This approach showed high efficiency for sc pDNA isolation, even using pDNA with different sizes, as well as for the separation of the linear isoform in sc pDNA samples. Moreover, to assess the efficiency of purifying sc pDNA, several samples with different oc percentages (ranging from 10–50%) were tested. It was shown that even with an oc/sc ratio of 1:1, the CIM^®^ pyridine was able to purify the sc isoform with a 95% homogeneity, following the FDA requirements. This that SDC is suitable for obtaining a pharmaceutical-grade sc pDNA, even from extracts with low levels of sc pDNA. Additionally, the SDC efficiency using the CIM^®^ monoliths was flow-independent and required a lower (NH_4_)_2_SO_4_ concentration than that commonly used for sample loading in HIC. Nevertheless, DBC for the pDNA was lower in SDC compared to the usual pDNA purification methods, but this disadvantage can be overcome by using continuous and multicolumn chromatographic systems [45].

Limonta et al. (2017) conducted a performance comparison between a CIM^®^ C4-HLD monolith, a high ligand density support with a strong hydrophobic ligand that can be used throughout the entire chromatographic procedure, and a Sartobind phenyl membrane column can be used in the purification of a pDNA vaccine against hepatitis C. The monolithic support showed the ability to duplicate the sc/oc ratio, yielding purities ranging from 92.8 to 99.4%, with host impurities within acceptable levels, and a recovery yield between 80.9 and 100%. This study confirmed the scalability of the monolith, enabling the production of a sufficient quantity of pharmaceutical-grade vaccine to initiate clinical trials [46].

##### Affinity Chromatography Using Monoliths for DNA Purification

Affinity chromatography (AC) is a type of liquid chromatography that utilizes a biologically based ligand in a chromatographic column to purify or analyze specific biomolecules based on reversible interactions. These specific interactions can arise from electrostatic forces, hydrophobic adsorption, van der Waals interactions, and/or hydrogen bonds. The unique characteristics of these interactions offer a considerable advantage in AC, contributing to its ability to achieve high selectivity and resolution in separating biomolecules [71]. Before the widespread use of monolithic supports, agarose-based stationary phases were effectively employed for pDNA purification by AC [72]. Alternative aromatic molecules, such as naphthalene tripodal, berenil, and 3,8-diamino-6-phenylphenanthridine (DAPP), have demonstrated their efficacy as ligands for the isolation of sc pDNA [73]. Berenil, in particular, exhibited a remarkable affinity for pDNA [74], demonstrating the ability to isolate pDNA from host impurities, independently of their size [5]. Notably, it also proved to be effective in negative chromatography, recovering a high-purity pDNA sample [75]. Similarly, porous supports utilizing DAPP [76,77,78] and naphthalene tripodal [79] ligands displayed significant promise for sc pDNA isolation. However, the limited binding capacity of these supports [76], as well as their high mass transfer resistance for large biomacromolecules like nucleic acids, hindered their industrial implementation.

In 2008, Han et al. pioneered the initial application of an affinity approach for purifying pDNA with monoliths. In this breakthrough, they utilized a 16-mer peptide mimicking the helix II of the DNA binding domain of the lac regulator protein as its ligand. The monolith was synthesized by polyethyleneglycol dimethacrylate (EDMA) and glycidyl methacrylate (GMA) polymerization, with subsequent 16-mer functionalization on the monolith matrix. Three supports with different column volumes (1, 4, and 5 mL monolith) were prepared to evaluate the scalability of the functionalization process, but only the 4 mL monolith was used for pDNA purification experiments. The results of peptide functionalization with the different monoliths showed that the immobilization kinetics were remarkably fast, completed in 30 min independently of the column volume, confirming the scalability of the coupling process. After validation with a sample of pure pDNA standard, the novel monolith successfully purified a clarified *E. coli* lysate, yielding a single pDNA peak in the purified fraction with an 81% recovery and 92% pDNA purity (A260/A280 ratio). Additionally, the effects of the NaCl concentration, residence time in the monolith, and flow rate, were evaluated for further optimization. The latter two parameters showed no significant effect on the binding between the 16-mer peptide and pDNA, while the NaCl concentration exhibited a stronger effect under IEX conditions rather than under HIC conditions. Therefore, the affinity capacity of the 16-mer ligand could be controlled by the concentration of NaCl. Finally, the potential reusability of this support was assessed over 16 consecutive runs, indicating no decline in performance over the stipulated lifetime [47].

Cernigoj et al. [48] employed a multimodal histamine-derivatized CDI monolith to separate pDNA isoforms from a clarified cell lysate, achieving a DBC range of 2.7 mg/mL to 4.0 mg/mL, according to the experimental conditions used. Because of that, the histamine monolith was used for purifying sc pDNA from cell lysates with high yield and purity. The affinity of histamine to the sc isoform and the increased DBC of monoliths [64] were leveraged in two purification strategies: a simple approach with an increasing NaCl gradient (resulting in 96.6% purity and 99.3% yield) and a combined strategy involving the same NaCl gradient with an immediate decreasing (NH_4_)_2_SO_4_ gradient (yielding 78.9% purity and 91.5% yield). Host impurity quantification revealed both strategies reduced all impurities below standard levels, except for host proteins, which were only undetectable with the combined strategy. The combined approach demonstrated superior efficiency, achieving a 128-fold and 39-fold reduction in endotoxins and gDNA, respectively [49].

Cardoso et al. (2015) pursued an alternative to HIC for isolating sc pDNA, aiming to improve upon the CIM^®^ C4 HDL monolith [50]. A preliminary screening with a phenyl ligand proved inefficient, leading to the introduction of a histamine monolith, capitalizing on its heteroaromatic ring structure. While enhancing isoform separation efficiency with a decreasing (NH_4_)_2_SO_4_ gradient, this modification sacrificed sc pDNA selectivity, prompting an increase in salt concentration in the elution buffer. To find a balance between column efficiency, salt concentration, and sc pDNA selectivity, a monolithic column with pyridine ligands was selected for comparison to the CIM^®^ C4 HDL. The new pyridine-based monolith exhibited a lower DBC (1.3 mg of sc pDNA/mL of support) compared to the CIM^®^ C4 HDL (1.8 mg of sc pDNA/mL of support). However, with an increase in (NH_4_)_2_SO_4_ concentration from 2 M to 3 M in the binding buffer, the new monolith surpassed the CIM^®^ C4 HLD, achieving a binding capacity of 3.2 mg of sc pDNA/mL of support. While both monoliths achieved recoveries exceeding 90%, the pyridine monolith demonstrated a resolution almost three times higher (1.7 oc/sc resolution factor) compared to the CIM^®^ C4 HDL (0.6 oc/sc resolution factor). Additionally, higher sc homogeneity and step yield were attained, with values of 98% and 96%, respectively. While the novel pyridine support showed promise as a potential CIM^®^ C4 HDL replacement in sc pDNA isolation, further adjustments are required to ensure compliance with FDA safety and quality regulations, particularly regarding the detection of host contaminants [50].

In 2013, Soares et al. devised a chromatographic affinity technique employing arginine as a ligand. They harnessed the exceptional selectivity of this amino acid, combined with the adaptability of CIM^®^ epoxy monoliths, to isolate sc pDNA from a pre-purified pDNA sample, a crucial step in producing the human papillomavirus vaccine. [80]. The arginine monolith showed a considerably higher DBC_10%_ (3.55 mg/mL) compared with the 0.18 mg/mL obtained with a conventional arginine–agarose matrix [80]. With this functionalized monolith, the purity sc pDNA fraction exceeded 99%, with a recovery yield of 39.2%. The impurity analysis indicated a reduction of host impurities to acceptable levels. However, this chromatographic procedure required further optimization to enhance the yield of the recovered sc pDNA fraction [51]. Following this work, Almeida et al. (2015) performed an optimization based on the design of experiments [52], identifying an optimal point with recovery and purity of 91.4% and 98.9%, respectively. In three chromatographic runs, a consistent purity of 100% was achieved, with a recovery yield of 75.8% to 88.8%, the latter slightly below the expected value. It is worth noting that impurity analysis of sc pDNA fraction was not performed, leaving the assessment of potential changes in the support’s capacity to remove host impurities unexplored [52]. Considering the successful application of histidine–agarose in separating oc and sc isoforms, Amorim et al. (2015) conducted a ligand screening with L-histidine derivatives, namely 1-methyl-L-histidine, 1-benzyl-L-histidine, and L-histidine, using surface plasmon resonance (SPR) [68]. The results indicated that L-histidine and 1-benzyl-L-histidine displayed the strongest affinity for various pDNA isoforms [53]. Building upon the arginine-based affinity monolith approach [51], this study introduced histidine-based affinity monoliths, driven by the pH-dependent hydrophobic imidazole ring and the versatile charge density of L-histidine. This modification aimed to improve versatility compared to L-arginine. Chromatographic studies involved decreasing (NH_4_)_2_SO_4_ concentration in step-elution mode using purified sc pDNA samples of different sizes as standards. The L-histidine monolith successfully purified this pDNA, achieving a DBC_10%_ of 4.2 mg pDNA/mL support while using a flow rate of 0.5 mL/min independently of pDNA size. The 1-benzyl-L-histidine monolith, used for the smallest pDNA, effectively also separated the sc pDNA isoform. Notably, this monolith required a lower (NH_4_)_2_SO_4_ concentration than the L-histidine monolith. This fact is aligned with SPR results, indicating a lower dissociation constant for the sc isoform with 1-benzyl-L-histidine. Agarose gel electrophoresis confirmed the purity of the fractions containing the sc pDNA peak [53]. The same authors also evaluated CIM^®^ L-histidine and CIM^®^ 1-benzyl-L-histidine in the purification of pDNA from a clarified *E. coli* lysate by manipulating pH (5 and 8). They observed different elution times for RNA and sc pDNA and, curiously, an inversion in the elution pattern occurred at pH 8, with sc pDNA eluting before RNA, suggesting hydrophobic-based separation. Considering the strategy with the elution at pH = 5 to separate RNA from pDNA, HPLC analysis revealed >99% sc purity in the sc pDNA peak for both supports. The L-histidine monolith achieved a 74.4% yield, while the 1-benzyl-L-histidine monolith only yielded 31.6%. Despite concerns about the acidic and high salt conditions affecting sc pDNA stability, a circular dichroism study demonstrated that pDNA reverted to its original conformation after the buffer exchange [54].

Cardoso et al. (2018) adopted a distinctive strategy employing arginine-based ligands for pDNA purification, utilizing a CIM^®^ epoxy monolith functionalized with arginine homopeptides. This innovative IEX ligand selection was grounded in the intracellular mechanisms triggered by arginine homopeptides, known to induce DNA condensation, thereby facilitating the delivery of these complexes to target cells [55]. Three distinct monoliths using as ligands arginine, di-arginine, and tri-arginine were employed. Post-experimental assessments revealed that the purity of pDNA samples, previously clarified with modified alkaline lysis and pre-purified on an IEX column, consistently fell below 97%, except for the tri-arginine ligand monolith that remarkably exceeded 99% purity. However, it was noted that the recovery yield exhibited a diminishing trend correlating with the ligand size, decreasing from 88.0% to 52.7%. Moreover, the analysis of potential impurities in the sc pDNA fraction affirmed the effectiveness of these monoliths in removing host impurities [51,55]. This study thus showcases the potential of arginine homopeptide-functionalized monoliths in pDNA purification, albeit with considerations regarding the trade-off between ligand size and recovery yield [81].

Bicho et al. [56] pursued an alternative strategy for purifying a hemagglutinin DNA influenza vaccine by utilizing a CIM^®^ polymethacrylate monolith functionalized with EDA. This ligand choice was rationalized based on the presence of amine groups at both ends of the molecule, facilitating ionic interactions between the negatively charged phosphate groups and the positive charges of the nitrogen in the amine groups. The outcome of this strategy yielded a sc pDNA fraction with 97.1% purity, aligning with FDA standards (>97%) [66]. Host impurity analysis substantiated a reduction to acceptable levels. However, the recovery yield stood at 47.0%, indicating there is room for refinement in this purification process [56]. Subsequently, Bicho et al. [57] explored a novel avenue for purifying the DNA influenza vaccine, employing a CDI monolith modified with the ligand agmatine. Agmatine is a product of arginine decarboxylation that exists endogenously in humans. This endogenous molecule was recently recognized for its potential therapeutic role in the treatment of alcohol addiction [82]. Agmatine differs from EDA by its guanidinium group in one of its ends, instead of an amino group. Thus, agmatine has a more positive net charge, which reinforces the interaction with the negative charges of the pDNA. The chromatographic process involved two different elution strategies: an increasing NaCl gradient and a decreasing (NH_4_)_2_SO_4_ gradient. With the former, two chromatographic runs yielded a purity of 99.6% and a recovery yield of 45.3%. Meanwhile, the latter strategy yielded a purity of 98.3% with a recovery yield of 51.8%. Given the relatively low recovery yields in both cases, it is recommended to explore further enhancements to the chromatographic protocol and/or the support material. Additionally, a DBC study revealed that lower flow rates, higher pDNA concentrations, and acidic conditions resulted in greater DBC on this monolithic support [57].

More recently, several chromatographic strategies based on monolithic supports and AC have been focused on the purification of mcDNA. This circular DNA marks a pivotal advancement in the evolution of pDNA pharmaceuticals, involving the recombination of parental plasmids (PP) into two smaller entities: the miniplasmid (MP), which possesses all the bacterial genes and the PP backbone, and the mcDNA that has the therapeutic gene cassette. The recombination outcome can either successfully yield the formation of MP and mcDNA or result in an unrecombined PP. On the other hand, this dual scenario complicates the pursuit of pharmaceutical-grade sc mcDNA, presenting a mixture of PP, MP, and mcDNA in the sample pool. To address some of these issues, Almeida and co-workers focused their work on optimizing new strategies based on monoliths for the purification and quantification of mcDNA. In 2019, they devised an analytical method employing a cadaverine-modified CIM^®^ monolith to accurately quantify sc mcDNA in complex *E. coli* lysates. Cadaverine, due to its terminal amine groups, facilitates ion interactions with pDNA phosphate groups and hydrophobic interactions. The method exhibited acceptable linearity, accuracy, and precision, with a lower limit of quantification and a threshold of detection of 1 μg/mL of mcDNA for a concentration range of 1–25 μg/mL of mcDNA. Robustness was validated through various mcDNA-PP pairs, confirming a consistent elution behavior. This cost-effective method was presented as a simpler alternative to the time/resource-intensive qPCR applications for mcDNA analysis [58]. In 2019, they evaluated individually a lysine and a cadaverine-based monoliths in purifying a 3.8 kbp mcDNA from *E. coli* lysates. The lysine-functionalized monolith demonstrated an inherent trade-off between recovery yield and purity, in addition to revealing a lack of selectivity for minicircular DNA. Conversely, the cadaverine ligand monolith proved to be effective, offering a recovery and purity of 78.6% and 98.4%, respectively, and successfully reduced impurities to levels compliant with FDA criteria [59]. In 2020, an epoxy monolith derivatized with arginine ligands was explored for purifying a DNA vaccine against human papillomavirus. The innovation in this work was the use of a spacer arm that enhances arginine ligand accessibility, thus increasing support sensitivity. Despite presenting a purity of 93.3%, slightly below regulatory standards, the monolith achieved a satisfactory recovery yield (72%). Impurity analysis indicated a reduction in host impurities to acceptable levels, but further assays should be performed to improve the purity percentage to at least 97% [60].

##### Other Monolith Supports for DNA Purification

In 2006, Wu et al. demonstrated the efficacy of microporous silica sol–gel-based microchip monoliths to separate DNA in diverse biological samples. These monoliths were prepared by mixing TMOS in PEG, followed by placement in the microchannel of the microchip and curing the sol–gel matrix. The microchip’s performance for isolating DNA from various sources, including λ-phage, human gDNA, cultured bacteria, and viral DNA from human spinal fluid, was evaluated. The results emphasize the potential applicability of microchip sol–gel devices as solid-phase monolithic supports for DNA extraction from diverse biological matrices. Notably, these devices exhibited remarkable extraction efficiencies and the capability to recover the target molecules into small elution volumes [61].

Holdšvendová et al. (2007) conducted an analytical study evaluating hydroxymethyl methacrylate-based monolith performance in the purification of mixed-sequence oligonucleotides of different sizes using hydrophilic interaction capillary liquid chromatography. The monoliths were synthesized using butane-1,4-diole and propane-1-ol as porogenic solvents, resulting in three different N-(hydroxymethyl) methacrylamide columns. After optimization of the buffer composition, a screened monolith achieved good resolution between 19- and 20-nucleotide oligonucleotidenucleotide oligonucleotide. Moreover, they demonstrated reproducibility from column to column in terms of retention time [62].

Smrekar et al. (2010) obtained pharmaceutical-grade pDNA by using an optimized CaCl2 precipitation, followed by a two-step purification combining AEX and HIC. This approach enabled the separation of sc from oc pDNA, achieving a final 98% purity and a 99% removal of gDNA and endotoxin, obtaining a final concentration below 2 EU/mg pDNA. The evaluation of the correlation between binding capacity and monolith pore size revealed an inverse relationship. A higher binding capacity was observed at lower pore sizes, although potential issues, including high back pressure and reduced diffusion mass transfer, can be observed at these conditions. The overall purification process demonstrated a process yield of 82%, processing 6 mg of pDNA, corresponding to a productivity of 2.2 mg pDNA/h. Additionally, reusability testing showed that these supports rend over 10 consecutive runs without sanitization and subsequent washing, without detriment in their separation and recovery capacity. Finally, the scalability of the process was also assessed, revealing a linear increase in the amount of pDNA processed and overall productivity with an eight-fold bed volume increase, while maintaining impurity removal capability [63]. Subsequently, Smrekar et al. (2013) developed a one-step approach to mimic the results of the previous two-step process [64] by introducing different functional groups on a single methacrylate monolith to exhibit both hydrophobic and ion-exchange interactions. Three monoliths were prepared: (1) Methacrylate monoliths bearing octylamine groups, (2) a hybrid monoltih incorporating butyl (C4) grafted methacrylate moieties alongside DEAE functional groups (gBuMA + DEAE), and (3) grafted chains with both C4 and DEAE groups (gBuMADEAE). Ionic capacity studies revealed the gBuMA + DEAE has the highest capacity, even surpassing a commercially available DEAE monolith. Despite the significantly low ionic capacity when compared to the commercial benchmark, the grafted DEAE monolith achieved the highest dynamic binding capacity (DBC) under HIC conditions. Octylamine monolith exhibited the lowest ionic capacity but the highest DBC under HIC conditions. The DBC for pDNA of both the octylamine and gBuMA + DEAE monoliths were tested under IEX and HIC binding conditions, as well as for their ability to separate sc and oc pDNA isoforms and RNA. gBuMA + DEAE provided a pDNA DBC of 2.1 and 4.7 mg of pDNA/mL of support under HIC and IEX conditions, respectively. The developed technique achieved over 99% removal of host cell proteins, RNA, and gDNA through a single-step purification process. Moreover, it exhibited the capability to purify approximately 1.5 mg of pDNA/mL of monolith from the initial sample, a level comparable to that achieved with a two-step monolith process [63]. Nevertheless, achieving the complete separation of both pDNA isoforms was not accomplished, necessitating further investigations to enhance this innovative monolithic column [64].

#### 2.1.2. RNA Purification

In recent years, the growing interest in RNA-based therapies, such as vaccines, cell reprogramming, protein replacement, and immunotherapy has prompted significant investments in innovative approaches for RNA production and purification. The high demand for mRNA, particularly in the context of COVID-19 vaccines, has required a technologically advanced and cost-effective manufacturing platform with a strict final product quality and safety standards. These vaccines are produced via in vitro transcription (IVT) followed by a multi-step purification procedure, which is normally carried out through chromatographic techniques. Similarly for other molecules, chromatography has been established as the preferential technique for RNA purification through various methodologies, as summarized in Table 2.

##### Ion Exchange Chromatography Using Monoliths for RNA Purification

A notable study by Krajacic et al. (2006) utilized a CIM^®^ DEAE anion exchange monolith to purify double-stranded (dsRNA) and satellite RNA (satRNA) produced by the Cucumber mosaic virus, a plant pathogen known to induce severe diseases in tomatoes. The researchers demonstrated that the CIM^®^ DEAE monolithic support serves as a practical and straightforward quantitative tool for assessing virus–satellite–host interactions. The method exhibited good reproducibility across different samples from various infection stages and plant growth phases, showing a distinct pattern of dsRNA and satRNA in the chromatogram [83].

In another application, presented by Perica et al. (2008), a DEAE-modified CIM^®^ monolith was utilized to purify hypoviral dsRNA. This practical approach for isolating hypoviral dsRNA, combined with a CF-11 purification step, offers a fast assessment without the need for additional steps to achieve the required purity. The combination of purification steps also facilitates the acquisition of a superior RT-PCR amplification template. Moreover, when screening fungal isolates, the application of a phenol/chloroform-extracted water phase application on the CIM^®^ DEAE monolith offers a reliable method for detecting hypoviral presence, eliminating the need for the CF-11 purification step [84].

In 2013, Romanovskaya et al. investigated two high-throughput dsRNA purification monoliths using a CIM^®^ as a matrix—one containing a strong AEC ligand (QA) and the other a weak AEC ligand (DEAE). Both monoliths outperformed a non-porous methacrylate resin with DEAE functionalization in terms of binding capacity. The DEAE-functionalized monolith achieved a noteworthy 8.0 mg dsRNA/mL resin, while the QA-functionalized monolith attained 5.5 mg dsRNA/mL resin. In contrast, the reference resin provided a considerably lower binding capacity of 0.6 mg dsRNA/mL resin. It is important to note that the DEAE-functionalized monolith exhibited a lower recovery rate (39%), contrasting with the QA-functionalized monolith and the reference resin, with a recovery of 52% and 55%, respectively [85].

Since conventional chromatographic supports do not provide proper conditions for laboratory-scale purification of oligonucleotides, Thayer et al. (2010) introduced an automated anion exchange purification technique for laboratory-scale oligonucleotide purification, addressing the limitations of conventional chromatographic supports. They utilized a monolithic support coated with nanobeads featuring a quaternary amine ligand (sAEX). This innovative support allowed flow rates of 3 mL/min and the nanobead coating minimized hydrophobic interactions and peak broadening and tailing, while enhancing selectivity. Comparative analysis with a benchmark revealed superior performance in terms of peak capacity, resolution, selectivity, and pressure stability. The sAEX monolith achieved oligonucleotide purity exceeding 90%, along with an 18% increase in yield compared to the benchmark porous column. This novel monolithic column effectively separated derivatized oligonucleotides from their unlabeled counterparts, various isobaric RNA linkage isomers, and phosphorothionate diastereoisomers in DNA and RNA. Notably, it enabled sample self-displacement purification of 8.25 mg of oligonucleotides in a single chromatographic run [86].

##### Other Monolith Supports for RNA Purification

In 2007, Satterfield et al. developed a microfluidic purification system using a photopolymerized monolith for the purification and pre-concentration of mRNA. The monomer solution includes GMA and ethylene glycol dimethacrylate. The porous polymer monolith was subsequently functionalized with a primary amine-terminated fluorescent dye or an oligo dT with a linked 5′NH_3_-C_6_. High-quality mRNA was used for the experiments. After purification, trapping optimization, and evaluating various monolithic microfluidic devices with different oligo-dT and locked nucleic acid (LNA) functionalization percentages, the authors achieved the best results with a monolith featuring an equal combination of oligo-dT and LNA. This configuration achieved an enrichment of 45.4-fold. However, when compared to the leading mRNA purification kit on the market, the device did not represent an improvement over the established product [87].

In 2014, Pereira et al. [88] aimed to develop mix-mode chromatographic support for purifying pharmaceutical-grade pre-miR-129. They utilized a CDI monolithic chromatographic support with an agmatine ligand, isolating RNA obtained from bacterial aliquots extracted using a modified acid guanidinium thiocyanate phenol–chloroform method [90]. Three chromatographic strategies—one with an increasing NaCl gradient, the other with an ascending arginine gradient, and the last with a descending (NH_4_)_2_SO_4_ gradient—were employed to enhance efficiency and selectivity. All methods enhanced the final recovery yield and purity of pre-miR-29. However, the approach utilizing the NaCl gradient yielded the highest recovery rate at 97.3%. Conversely, the arginine-based strategy exhibited the highest purity (90.1%). Host impurity analysis of the purified pre-miR-29 fraction confirmed the monolith’s ability to isolate the analyte without significant impurities [88].

In 2018, Levanova et al. utilized CIM^®^-OH monolithic columns based on steric exclusion chromatography to purify dsRNA and single-stranded (ssRNA). These RNAs were enzymatically synthesized and isolated with TRIzure reagent and chloroform. The results demonstrated the support’s capability to separate dsRNA from ssRNA, providing greater resolution for dsRNA larger than 700 bp. CIM^®^-OH monolith also successfully purified entire viral ssRNA and dsRNA genomes from contaminants. However, this method presents some limitations in isolating RNAs with diverse sizes (88 bp to 6374 bp), only effectively separating short RNA fragments (<100 bp) from longer RNA [89].

## 3. Cryogels

Cryogels represent a novel class of supermacroporous chromatographic supports characterized by a sponge-like morphology and a network of channels with controlled pore size [89]. Originally reported in the 1970s, these polymeric gels intrigued researchers due their unique properties [91]. One major advantage of cryogels compared to traditional chromatographic supports is related to mass transfer. Cryogels ensure unrestrictive convective transport, whereas the latter can only perform mass transport through diffusion [92]. As a result, cryogels facilitate the efficient separation of nanoparticles, cellular organelles, and even whole cells via chromatographic methods [93]. The convective flow, as depicted in Figure 2, is exclusively facilitated by the macropores inherent in the cryogels, ranging in size from 10 to 100 µm [94].

Cryogenic monoliths represent promising alternatives to conventional protein-binding matrices, finding diverse biomedical applications [95] which include affinity-based approaches [96,97,98,99], molecular imprinting [100,101,102,103,104,105,106], dye-affinity [107,108] and others employed for processing proteins such as human serum albumin [106] and immunoglobin G [109]. The advantages of cryogenic monoliths, including high blood-compatibility and water content, non-degradability, absence of toxicity, and favorable pressure drop properties, make them suitable for the purification of biological macromolecules without diffusion issues [96]. Another strong point of cryogels is their unique and tunable properties, which makes them valuable in various fields through the control of fabrication parameters (e.g., polymer choice, temperature, solute concentration, and cooling rate) [94], as well as their mechanical stability ad elasticity that reinforces their adaptability to be used in a wide range of applications [110].

Applications for cryogel support are divided into four categories: capture and purification of biomolecules, biomolecule immobilization, separation of cells, and environmental separations [91]. This review primarily focuses on the first category. Additionally, cryogels can be modified for use in research that focuses on tissue engineering. Because of its three-dimensional structures, hydrophilicity, and a direct positive impact on cell migration, proliferation, and differentiation, and cryogels have emerged as highly promising tools for a variety of biomedical applications [95].

### 3.1. Preparation of Cryogels

**Figure 3:**
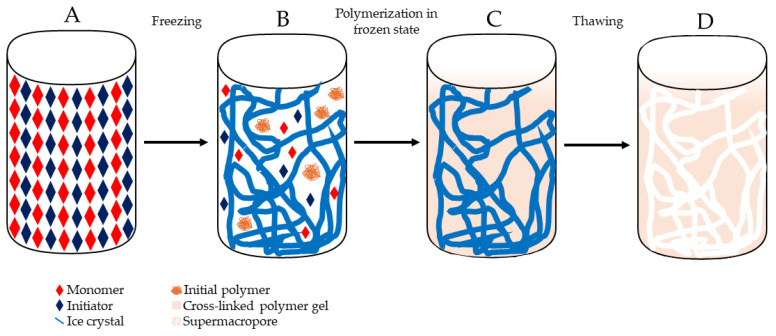
Cryogelation process. A solution of monomers and initiators is prepared in a cryogelation solvent and then is frozen (**A**), resulting in the formation of ice crystals (**B**). During freezing, an unfrozen liquid microphase (UFLMP) is formed, trapping polymer precursors, and allowing crystallization to progress, forming a network (**C**). Thawing the mixture at room temperature causes the ice to melt leaving behind macropores surrounded by a gel matrix and forming a supermacroporous interconnected structure (**D**).

Cryogels are prepared by a process known as cryogelation, taking place at temperatures ranging from −5 to −20 °C, depending on the solvent crystallization point [92]. This process involves three key steps, as illustrated in Figure 3. In the initial step, a solution comprising monomers and initiators is prepared, typically utilizing water as the solvent, and subsequently subjected to freezing. This stage comprises two primary components: the frozen solvent, which leads to the formation of ice crystals, and the unfrozen liquid microphase (UFLMP), where the polymer precursors are entrapped. With the progression of the crystallization process, the ice crystals connect, forming a cohesive network. Since the UFLMP constitutes only a small fraction of the starting volume, the concentration of gel precursors increases exponentially, facilitating gel formation around the crystals. After polymerization, the solution warmed to room temperature, causing the crystals to melt. This process leaves behind macropores surrounded by the newly formed cryogel, resulting in support with a complex system of macroporous [89,94].

By using different types of monomers such as pre-made vinylic monomers, 2-hydroxyethyl methacrylate (HEMA) [111] or acrylamide [112], cryogel chromatographic supports can be created. In addition, polymers with natural or synthetic character, such as alginate [113], agarose [113], gelatin [114], or chitosan [115], can be modified via free radical cryo-polymerization with vinylic groups to cross-link them. An APS and TEMED initiator system is the most commonly used for cryogel fabrication [94].

### 3.2. Cryogels for Nucleic acid Purification

Cryogel-based supports were initially employed as chromatographic platforms for purifying proteins and other biological macromolecules. Due to their enhanced biocompatibility compared to monolithic supports [116], and the presence of pores that facilitate the passage of larger molecules like pDNA and other nucleic acids, cryogels have the potential to replace monolith-based chromatographic supports in the purification of these biomolecules.

#### 3.2.1. DNA Purification

The performance of several cryogel supports in DNA purification has been assessed, as detailed below, and summarized in Table 3.

Efficient capture of pDNA directly from non-clarified cell lysate was achieved by Hanora et al. (2006) using macroporous polyacrylamide grafted with polycations demonstrated grafting degrees ranging from 34% to 110%. These polycation-grafted monolithic columns effectively captured pDNA directly from alkaline *E. coli* lysates. The large pore size in these macroporous monoliths prevented blockage by particulate material in non-clarified feeds. Elution of the captured pDNA was achieved by using 1 M NaCl, resulting in a particles eluate with a greatly decreasing in protein and RNA content compared to the applied lysate [117]. This approach was also demonstrated using monolithic columns from macroporous polyacrylamide in a 96-well microtiter plate format, suggesting a potentially attractive technique for high-throughput screening of pDNA purification from several production systems [122].

In 2011, Perçin et al. introduced cryogel technology for purifying pDNA, employing a poly(hydroxyethyl methacrylate-N-methacryloyl-(L)-histidine methyl ester) (PHEMAH) cryogel for the purification of pDNA from *E. coli* lysates. The pseudo-affinity ligand N-methacryloyl-(L)-histidine methyl ester was synthesized from L-histidine methyl ester and methacryloyl chloride and the quality of the synthesis was confirmed by H^1^ NRM analysis. The cryogel was produced through free-radical polymerization in an ice bath, using TEMED and APS as initiators. The capability of PHEMAH cryogel to purify pDNA from a clarified *E. coli* lysate was assessed, as well as their reusability. The results demonstrated that the novel cryogel is capable of binding to isolate pDNA to a great extent, achieving a binding capacity of 13.5 mg of pDNA/g of polymer. However, purity analysis by electrophoresis revealed that the majority of the pDNA is in its linear isoform. Furthermore, this cryogel maintained its binding capacity above 10 mg of pDNA/g of polymer even after three reuse cycles [118].

Üzek et al. [119] conducted a study aiming to enhance the cryogelation process by incorporating nanospines into the cryogel structure. Three distinct cryogels were created: a poly(2-hydoxyethyl methacrylate-N-methacryloyl-l-phenylalanine p(HEMA-MAPA) cryogel, a poly(2-hydoxyethyl methacrylate-N-methacryloyl-l-phenylalanine)-freeze dried [p(HEMA-MAPA)-FD] cryogel, and a poly(2-hydoxyethyl methacrylate)-freeze dried [p(HEMA)-FD] cryogel. The surface area of all of the supports was evaluated via the Fourier-transform infrared spectroscopy (FTIR) technique, scanning electronic microscope (SEM), and nitrogen adsorption/desorption with the Brunauer–Emmett–Teller isotherm. Studies for the adsorption of DNA were done to analyze the effects of pH, temperature, salt type and concentration, and initial pDNA concentration. Optimal conditions were established for DNA adsorption, including a pH of 5–6, a temperature at 40 °C, 1 M sodium sulfate, and 4 mg/mL of DNA for the loading conditions. The [p(HEMA-MAPA)-FD] support presented superior DBC value, reaching 45.31 mg of DNA/g of cryogel—two times higher than a cryogenic support prepared through conventional methods and 40 times greater than poly(HEMA) (pHEMA) cryogels. After the DBC experiments, pDNA purification was performed, revealing a decrease in the amount of oc isoform in the elution peak, accompanied by an increase in the intensity of the sc isoform band in electrophoresis. Unfortunately, an assessment of host impurities was not conducted, therefore it was not determined if the purified samples align with safety regulations [119].

Çorman et al. (2013) also synthesized two alternative hydrophobic cryogels designed for isolating gDNA from Salmon tissue pre-clarified using phenol–chloroform (1:1) extraction, followed by a 70% ethanol precipitation. The developed supports included a pHEMA-N-methacryloyl-L-tryptophan) cryogel with methacryloyl-L-tryptophan hydrophobic ligands (pHEMA-MATrp) and a pHEMA cryogel embedded with pHEMA-MATrp monosize particles [89]. APS and TEMED were used as initiators, for the synthesis of both of the cryogels used. Their assessment was executed with the use of the FTIR spectroscopy technique, swelling studies, SEM microscopy, surface area measurements, and elemental analysis. This study also demonstrated that DNA was strongly adsorbed by specific hydrophobic interactions at pH 5.0. Furthermore, salt concentration studies determined that the use of sodium sulfate contributes to a higher DNA adsorption to the supports. The pHEMA-MATrp cryogel exhibited a maximum DNA adsorption of 15 mg/g of polymer, while the cryogel with HEMA-MATrp monosize particles achieved a higher maximum DNA adsorption of 38 mg/g of polymer [89].

In 2015, the same research team introduced a pHEMA cryogel containing dye affinity ligands for the purification of pDNA from *E. coli* lysates [120]. Free radical polymerization with the APS and TEMED initiators was used to produce the pHEMA support, followed by the incorporation of Cibacron Blue F3GA to function as the affinity ligand. SEM and FTIR spectroscopy were used for characterization and the effects of the ionic strength of the salts used, the temperature, and the concentration of DNA on the purification process were investigated. The results indicated that low temperatures and ionic strength, and high DNA concentrations, were the optimal performance parameters for this chromatographic support. Notably, a DBC of 32.5 mg/g of cryogel was achieved, marking a nearly 30-fold increase compared to the unfunctionalized cryogel [120].

More recently, Santos et al. (2018) synthetized a pHEMA support via cryo-polymerization with later analysis with SEM microscopy to perform the purification of an influenza DNA vaccine. The DBC of the cryogel was also assessed, revealing a DBC_10%_ range from 0.010 to 0.142 mg of pDNA/g of cryogel, depending on the loaded pDNA concentration. The low DBC values were correlated with the absence of ligands on the cryogel’s surface. By using a two-step purification method, isolation of the sc pDNA isoform from a clarified lysate sample was accomplished with NaCl-based elution. Evaluation of the DNA vaccine confirmed compliance with FDA regulations regarding the presence of contaminants such as proteins, gDNA, RNA, and endotoxins. The overall process yielded a purity of 98.1% and a recovery yield of 69.2%. A noteworthy aspect of this study is that it proves the specificity of cryogel support to scDNA, highlighting its potential for sc pDNA purification when coupled with a high-selectivity ligand [111].

Immobilized metallic chelate affinity (IMAC) technology was implemented into cryogel supports for pDNA isolation from a lysate sample by Önal and Odabaşı (2021), where an Fe^3+^, extracellular polymeric substances, and PHEMA composite cryogel were used. After optimization, a binding capacity of 39.7 mg of pDNA/g of cryogel was obtained, as well as an Abs260/280 ratio of 1.85, indicating that the eluted DNA has a high degree of purity. Even though this novel affinity approach provides a high binding capacity, further studies need to be conducted to evaluate its performance (purity and recovery) for sc isolation and impurity (RNA, host proteins, gDNA, endotoxins) removal [121].

#### 3.2.2. RNA Purification

The use of cryogels previously applied for DNA purification has been expanded to RNA purification in the hope of replicating the success achieved in DNA purification. Table 4 lists the cryogenic supports used with the objective to purify RNA.

In 2012, Srivastava et al. utilized a Poly(Hydroxyethyl Methacrylate-Co-Vinyl Phenyl Boronic Acid) cryogel for RNA separation from *E. coli* extracts in a single step. DBC for this support was quantified at 1.1 mg/mL. Despite demonstrating successful RNA separation from bacterial extracts, the possible impurity evaluation for the purified RNA samples was not presented [123].

Köse et al. (2016) introduced an innovative cryogel designed for the isolation and purification of RNA [124]. They synthesized a polymerizable derivative of adenine, denoted as adenine methacrylate, through a substitution reaction where adenine and methacryloyl chloride were the interveners. Using adenine methacrylate and HEMA monomers, they prepared HEMA-based cryogels in a partially frozen medium through copolymerization. Batch system experiments were conducted under various conditions of pH, initial RNA concentration, temperature, and interaction time. The resulting cryogel exhibited a swelling ratio of 510%, a capacity of 11.9 mg/g, and the ability to be reused up to five times [124]. This research group further pioneered the creation of a new cryogel featuring a pHEMA matrix incorporated with guanine. Their aim was to attain highly purified RNA by leveraging the inherent interaction between guanine within the polymeric material and cytosine in RNA. This cryogel was created by using HEMA and GuaM monomers with a TEMED and APS combination for initiators, demonstrated superior adsorption capacity (5.6 mg/g) compared to a standard pHEMA cryogel and a commercial kit. The novel cryogel exhibited and DBC value that is almost 29 times higher than the unmodified support, but also 1.6 times higher than the commercial kit used as benchmark. The natural interaction facilitated highly selective adsorption in a small amount of time with no diffusion issues. Moreover, RNA adsorbed onto this new cryogel could be efficiently recovered without denaturation, enabling fast, cost-effective, and single-step RNA purification. While the removal of impurities from crude samples was not explicitly demonstrated, the novel cryogel showed promising results in selective and efficient RNA purification [125].

## 4. Conclusions

In recent years, the need for nucleic acids has been steadily increasing, driven by their applications in gene therapy and vaccine development. The obtention of these biopharmaceuticals via biotechnological processes has attempted to suppress this demand. Nevertheless, the purification step remains a main challenge since nucleic acids required for these biomedical applications should meet the regulatory agencies’ demands.

Chromatography is a purification platform commonly used in the pharmaceutical industry; however conventional chromatographic supports used for nucleic acid purification exhibit some drawbacks, such as low binding capacity and issues during the scale-up process. To mitigate these limitations and enhance the efficiency of the purification process, continuous beds have emerged as a novel alternative to conventional chromatographic methods. The manufacturing of these continuous bed supports is highly dynamic, which means that different materials and ligands could be used, allowing them to have unique features that are appealing for the separation and isolation of target biopharmaceuticals, such as DNA and RNA. However, cryogels and monoliths also present some limitations that need to be addressed. Despite their advantages, cryogels may exhibit a lower surface area compared to monoliths, potentially impacting binding capacity in specific applications. Additionally, while these supports minimize mass transfer resistance, they still can exhibit some diffusion transport, which can affect the efficiency of separations, especially in complex matrices. Fouling and clogging are other issues encountered in chromatographic columns utilizing these continuous supports, leading to reduced dynamic binding capacity and increased pressure drop across the system, ultimately affecting its longevity and performance.

Although it has been demonstrated that these matrices can serve as viable alternatives to packed beds for the efficient purification of nucleic acids at an industrial scale, monolithic and cryogel supports with better performance parameters should be carried out to assure their up-scaling. These challenges underscore the importance of further research and optimization to address scaling issues, ensure environmental sustainability, and enhance the efficiency of chromatographic purification methods using monoliths and cryogels. Nevertheless, recent advancements in chromatography using monolithic and cryogel-based supports for nucleic acid purification have significantly improved process efficiency and specificity due the high porosity, increasing permeability and reducing back pressures in chromatographic systems. These continuous-bed systems also offer versatility in ligands and formats, adaptable for use in various configurations such as columns, disks, capillaries, and microchips. This versatility allows tailored purification processes based on specific requirements. By leveraging these advancements, researchers have significantly improved the speed, throughput, versatility, and selectivity of nucleic acid purification methods. This progress enables more efficient and specific isolation of nucleic acids for various downstream applications, offering versatility in formats and ligands, allowing for tailored purification processes based on specific requirements.

## Figures and Tables

**Table 1:** Summary of different monolithic supports used for DNA purification.

Target	Ligand	Matrix	Binding Capacity (mg/mL)	Purity (%)	Recovery Yield (%)	Application	Reference
pDNA	DEAE	CIM^®^	8.9	92.0	100.0	-	[39]
gDNA	DEAE	CIM^®^	-	-	-	-	[40]
pcDNA3F	Triethylamine	Poly (GMA-EDMA)	15.8	-	>95.0	-	[41]
	Diethylamine		21.5				
pDNA	DEAE and QA	CIM^®^		-	82.0–100.0	-	[42]
G-quadruplex	QA	CIMmultus^TM^	5.5	92.0	-	-	[43]
pDNA	-	CDI	-	100.0	74.0	-	[44]
pDNA	Pyridine	CIM^®^	0.5	>95.0	-	-	[45]
pDNA	C4 HLD	CIM^®^	0.9–2.0	92.8–99.4	80.9–100.0	Hepatitis C DNA vaccine	[46]
pDNA	16mer (oligonucleotide)	poly (GMA-co-EDMA)	0.02	92.0	81.0	-	[47]
pDNA	Histamine	CDI	4.0	98.5	97.0	-	[48]
pDNA	Histamine	CDI	2.7–4.0	78.9–96.7	91.6–99.3	-	[49]
pDNA	Pyridine	CIM^®^	1.3	>90.0	98.0	-	[50]
pDNA	Arginine	CIM^®^ epoxy	5.2	>99.0	39.2	HPV DNA vaccine	[51]
pDNA	Arginine	CIM^®^ epoxy	-	100.0	75.8–88.8	HPV DNA vaccine	[52]
pDNA	L-Histidine	CIMac^TM^	11.0	-	-	Ligand screening	[53]
	1-benzyl-L-Histidine		-	-	-		
pDNA	L-Histidine	CIMac^TM^	2.4	>99.0	74.4	-	[54]
	1-benzyl-L-Histidine		-		31.6	-	
pDNA	Arginine	CIM^®^ epoxy	1.5	>97.0	88.0	-	[55]
	di-Arginine		3.5		66.1	-	
	tri-Arginine		3.6	>99.0	52.7	-	
pDNA	Ethylenediamine	CIM^®^ polymethacrylate	-	97.1	47.0	Influenza DNA vaccine	[56]
pDNA	Agmatine	CDI	8.6	99.8	98.3–99.6	Influenza DNA vaccine	[57]
mcDNA	Cadaverine	CIM^®^	-	-	-	Analytical approach	[58]
mcDNA	Lysine	CIM^®^ epoxy	-	-	-	-	[59]
	Cadaverine		2.4	98.4	78.6		
pDNA	Arginine + Space arm	Epoxy	2.5	93.3	72.0	HPV DNA vaccine	[60]
DNA	-	TMOS + PEG	-	-	-	Separation of several DNA types	[61]
Oligonucleotides	-	HMMAA-(EDMA)	-	-	-	-	[62]
pDNA	DEAE and C4	CIM^®^ OH (hydroxy)	-	98.0	81.0	-	[63]
pDNA	Octylamine (IEX conditions)	CIM^®^ epoxy	1.6	-	>80.0	-	[64]
	Octylamine (HIC conditions)		0.6	-			
	gBuMA + DEAE (IEX conditions)		4.7	-			
	gBuMA + DEAE (HIC conditions)		2.1	-			

Abbreviations: Butyl (C4); Carbonyldiimidazole (CDI); convective interaction media (CIM^®^); Diethylaminoethyl (DEAE); genomic DNA (gDNA); Glycidyl methacrylate (GMA); human papillomavirus (HPV); minicircle DNA (mcDNA); N-(hydroxymethyl) methacrylamide (HMMAA); quaternary ammonium (QA); plasmid pDNA (pDNA); Polyethyleneglycol Dimethacrylate (EDMA); Polyethylene Glycol (PEG); Tetramethoxysilane (TMOS).

**Table 2:** Summary of different monolithic supports used for RNA purification.

Target	Ligand	Matrix	Binding Capacity	Purity (%)	Recovery Yield (%)	Application	Reference
satRNA and dsRNA	DEAE	CIM^®^	-	-	-	detection and identification	[83]
dsRNA	DEAE	CIM^®^	-	-	-	-	[84]
dsRNA and siRNA	DEAE	CIM^®^	8.0 mg dsRNA/mL resin	-	39.0	-	[85]
	Quaternary amine		5.5 mg dsRNA/mL resin	-	52.0		
Oligonucleotides	Nanobeads w/Quartenary amine	ProSwift SCX-1S	-	>90.0	75.0	-	[86]
mRNA	Oligo dT	EGDMA-(GMA)	-	-	-	-	[87]
Pre-miR-29	Agmatine	CDI (NaCl gradient)	8.1 mg RNA/mL support	75.2	97.3	-	[88]
	Agmatine	CDI (Arginine gradient)		90.1	94.9	-	
RNA	OH (hydroxy)	CIM^®^	-	-	80.0	-	[89]

Abbreviations: Carbonyldiimidazole (CDI); convective interaction media (CIM^®^); Diethylaminoethyl (DEAE); double-stranded RNA (dsRNA); Ethylene glycol dimethacrylate (EGDMA); Glycidyl methacrylate (GMA); satellite RNA (satRNA); small interfering RNA (siRNA); Sodium chloride (NaCl).

**Table 3:** Summary of different cryogel supports used for DNA purification.

Target	Ligand	Matrix	Binding Capacity (mg/g)	Purity (%)	Recovery Yield (%)	Application	Reference
DNA	p(HEMA-MATrp) monosize particles	pHEMA	38.0	-	-	-	[89]
pDNA	-	pHEMA	-	98.1	69.2	Influenza DNA vaccine	[111]
pDNA	Polycations	Polyacrylamide	-	-	-	-	[117]
pDNA	MAH	pHEMA	-	90.0	-	-	[118]
DNA	MATrp	pHEMA-MATrp	15.0	-	-	-	[119]
pDNA	Cibacron Blue F3GA	pHEMA	32.5	-	-	-	[120]
pDNA	Fe^3+^ and EPS	pHEMA	39.7	-	-	-	[121]

Abbreviations: extracellular polymeric substances (EPS); Poly(2-Hydroxyethyl Methacrylate) (pHEMA); Poly(2-hydroxyethyl methacrylatep-N-methacryloyl-L-tryptophan (pHEMA-MATrp); plasmid DNA (pDNA).

**Table 4:** Summary of different cryogel supports used for RNA purification.

Target	Ligand	Matrix	Binding Capacity	Application	Reference
RNA	Boronate	PHEMA-co-VPBA)	1.1 mg/mL cryogel	-	[123]
RNA	Adenine	pHEMA	11.9 mg/g cryogel	-	[124]
RNA	Guanine	pHEMA	5.6 mg/g cryogel	-	[125]

Abbreviations: Poly(2-Hydroxyethyl Methacrylate) (pHEMA); Poly(Hydroxyethyl Methacrylate-Co-Vinyl Phenyl Boronic Acid (PHEMA-co-VPBA).

## Data Availability

Not applicable.

## Abbreviations

(NH_4_)_2_SO_4_	Ammonium Sulfate
AC	Affinity chromatography
AEX	Anion exchange chromatography
APS	Ammonium Persulfate
C4	Butyl
CDI	Carbonyldiimidazole
CIM^®^	Convective interaction media
DAPP	3,8-Diamino-6-Phenylphenanthridine
DBC	Dynamic binding capacity
DEAE	Diethylaminoethyl
dsRNA	Double-stranded RNA
EDA	Ethylenediamine
EDMA	Polyethyleneglycol Dimethacrylate
FDA	Food and Drug Administration
G4s	Guanine Quadruplexes
gDNA	Genomic DNA
GMA	Glycidyl Methacrylate
HEMA	2-Hydroxyethyl Methacrylate
IEX	Ion exchange chromatography
mcDNA	Minicircle DNA
mRNA	Messenger RNA
NaCl	Sodium Chloride
oc	Open circular
pDNA	Plasmid DNA
PEG	Polyethylene Glycol
pHEMA	Poly(2-Hydroxyethyl Methacrylate)
PHEMAH	Poly(Hydroxyethyl Methacrylate-N-Methacryloyl-(L)-Histidine Methyl Ester
pHEMA-MATrp	Poly(2-hydroxyethyl Methacrylate)-N-methacryloyl-L-tryptophan
PP	Parental plasmids
QA	Quaternary Ammonium
satRNA	Satellite RNA
sc	Supercoiled
SDC	Sample displacement chromatography
ssRNA	Single-stranded
TEMED	N,N,N,N-tetramethylethylenediamine
TMOS	Tetramethoxysilane
UFLMP	Unfrozen liquid microphase
